# *catena*-Poly[[[bis­(methanol-κ*O*)bis­(seleno­cyanato-κ*N*)manganese(II)]-μ-1,2-bis­(pyridin-4-yl)eth­ene-κ^2^ *N*:*N*′] 1,2-bis­(pyridin-4-yl)eth­ene mono­solvate]

**DOI:** 10.1107/S1600536813012609

**Published:** 2013-05-15

**Authors:** Susanne Wöhlert, Inke Jess, Christian Näther

**Affiliations:** a Institut für Anorganische Chemie, Christian-Albrechts-Universität Kiel, Max-Eyth-Strasse 2, 24118 Kiel, Germany

## Abstract

In the crystal structure of the title compound, {[Mn(NCSe)_2_(C_12_H_10_N_2_)(CH_3_OH)_2_]·C_12_H_10_N_2_}_*n*_, the Mn^II^ cation is coordin­ated by two terminal *N*-bonded seleno­cyanate anions, two methanol mol­ecules and two 1,2-bis­(pyridin-4-yl)eth­ene (bpe) ligands within a slightly distorted octahedral geometry. The Mn^II^ cations are linked into chains along the *c*-axis direction by the bpe ligands, which are further connected by inter­molecular O—H⋯N hydrogen bonding between the methanol H atoms and additional bpe mol­ecules that are not coordinated to the metal atoms. The Mn^II^ cation and both crystallographically independent bpe ligands are located on centers of inversion, whereas the seleno­cyanate and methanol ligands occupy general positions.

## Related literature

For background to this work see: Boeckmann & Näther (2010 ▶, 2012 ▶); Wöhlert *et al.* (2012 ▶). 
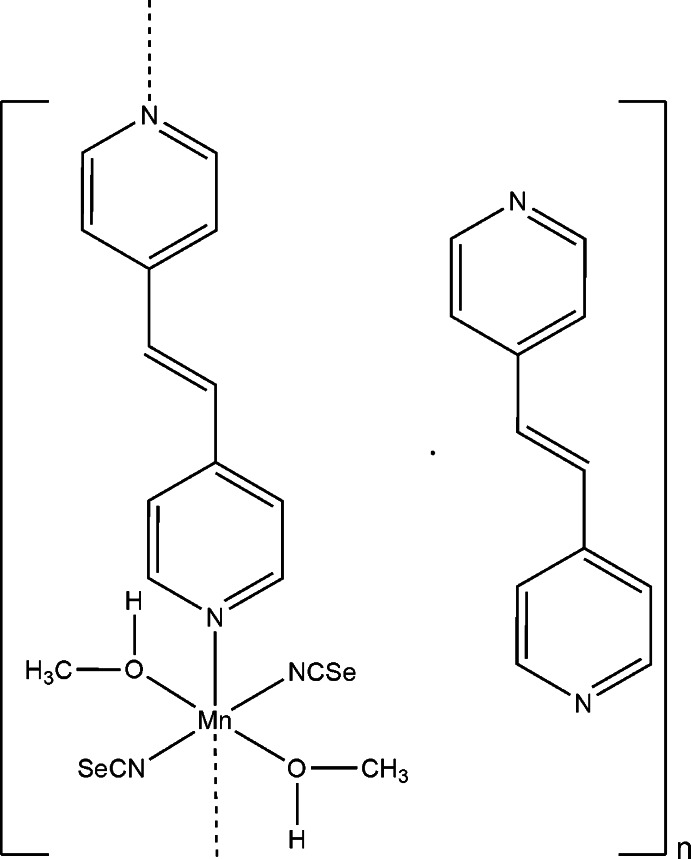


## Experimental

### 

#### Crystal data

[Mn(NCSe)_2_(C_12_H_10_N_2_)(CH_4_O)_2_]·C_12_H_10_N_2_
*M* *_r_* = 693.42
Monoclinic, 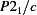

*a* = 7.3580 (6) Å
*b* = 17.2445 (11) Å
*c* = 12.1219 (9) Å
β = 92.630 (9)°
*V* = 1536.5 (2) Å^3^
*Z* = 2
Mo *K*α radiation
μ = 2.83 mm^−1^
*T* = 220 K
0.13 × 0.08 × 0.05 mm

#### Data collection

Stoe IPDS-1 diffractometer
Absorption correction: numerical (*X-SHAPE* and *X-RED32*; Stoe & Cie, 2008 ▶) *T*_min_ = 0.754, *T*_max_ = 0.862
14170 measured reflections
2633 independent reflections
2072 reflections with *I* > 2σ(*I*)
*R*_int_ = 0.091

#### Refinement

*R*[*F* ^2^ > 2σ(*F* ^2^)] = 0.041
*wR*(*F* ^2^) = 0.112
*S* = 0.99
2633 reflections
179 parameters
H-atom parameters constrained
Δρ_max_ = 0.38 e Å^−3^
Δρ_min_ = −0.78 e Å^−3^

### 

Data collection: *X-AREA* (Stoe & Cie, 2008 ▶); cell refinement: *X-AREA*; data reduction: *X-AREA*; program(s) used to solve structure: *SHELXS97* (Sheldrick, 2008 ▶); program(s) used to refine structure: *SHELXL97* (Sheldrick, 2008 ▶); molecular graphics: *XP* in *SHELXTL* (Sheldrick, 2008 ▶) and *DIAMOND* (Brandenburg, 2011 ▶); software used to prepare material for publication: *XCIF* in *SHELXTL* and *publCIF* (Westrip, 2010 ▶).

## Supplementary Material

Crystal structure: contains datablock(s) I, global. DOI: 10.1107/S1600536813012609/zl2549sup1.cif

Structure factors: contains datablock(s) I. DOI: 10.1107/S1600536813012609/zl2549Isup2.hkl

Additional supplementary materials:  crystallographic information; 3D view; checkCIF report

## Figures and Tables

**Table 1:** Selected bond lengths (Å)

Mn1—N1	2.185 (3)
Mn1—O1	2.188 (2)
Mn1—N10	2.281 (2)

**Table 2:** Hydrogen-bond geometry (Å, °)

*D*—H⋯*A*	*D*—H	H⋯*A*	*D*⋯*A*	*D*—H⋯*A*
O1—H1*O*1⋯N30	0.83	1.85	2.675 (4)	173

**Fig. 1.**
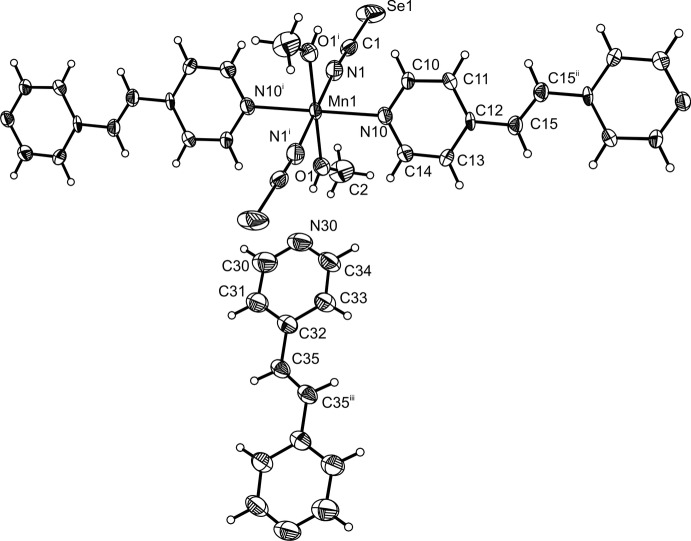
: Crystal structure of the title compound with labeling and displacement ellipsoids drawn at the 50% probability level. Symmetry codes: i = -x+2, -y+1, -z+1; ii = -x+1, -y+1, -z; iii = -x+2, -y, -z+1.

**Fig. 2.**
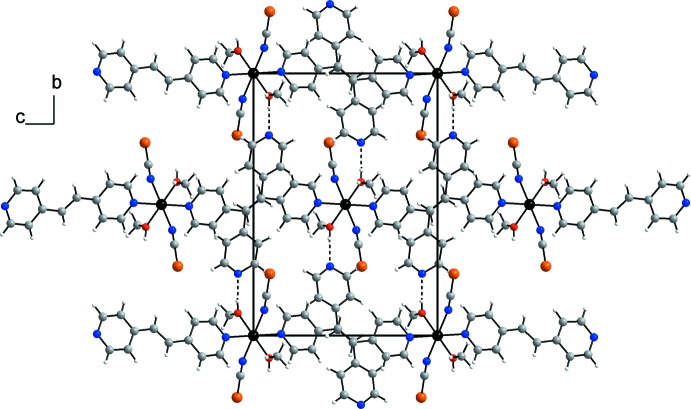
: Crystal structure of the title compound with view along the a-axis (black = manganese, blue = nitrogen, orange = selenium, red = oxygen, grey = carbon, white = hydrogen). Intermolecular hydrogen bonding is shown as dashed lines.

## supplementary crystallographic information

### Comment

Recently, we have reported on the synthesis, thermal and magnetic properties of new coordination polymers based on paramagnetic transition metal thiocyanates with different neutral co-ligands like e. g. pyridine, 1,2-bis(pyridin-4-yl)ethene (Boeckmann & Näther, 2010, 2012; Wöhlert *et al.*, 2012). In the course of these investigations we have reacted manganese(II) chloride dihydrate with potassium selenocyanate and 1,2-bis(pyridin-4-yl)ethene in methanol, which leads to the formation of crystals of the title compound that were identified by single-crystal structure analysis.

In the crystal structure of the title compound each manganese(II) cation is coordinated by two terminally *N*-bonded selenocyanate anions, two methanol molecules and two 1,2-bis(pyridin-4-yl)ethene (bpe) ligands within slightly distorted octahedra (Fig. 1). The Mn—O and Mn—N distances range from 2.187 (3) Å to 2.279 (3) Å with angles arround the manganese(II) cation between 86.82 (11) ° and 93.18 (11) ° and 180 ° (Tab. 1). The Mn(II) cations are linked by the bpe ligands into chains, which elongate in the direction of the crystallographic *c*-axis (Fig. 2). These chains are further linked into layers by intermolecular O—H···N hydrogen bonding to the non-coordinated bpe ligands (Fig. 2, Tab. 2).

### Experimental

MnCl_2_×2H_2_O, KNCSe and 1,2-bis(pyridin-4-yl)ethene were obtained from Alfa Aesar. All chemicals were used without further purification. 0.15 mmol (24 mg) MnCl_2_×2H_2_O and 0.2 mmol (28 mg) KNCSe were reacted with 0.3 mmol (54 mg) 1,2-bis(pyridin-4-yl)ethene in 1 ml methanol.

### Refinement

All C—H atoms were positions with idealized geometry (methyl H atoms allowed to rotate but not to tip) and were refined isotropic with *U*_iso_(H) = 1.2 *U*_eq_(C) using a riding model with C—H = 0.94 and 0.97 Å. The O—H H atom was located in a difference map, its bond length was set to 0.83 Å, and finally it was refined isotropically with *U*_iso_(H) = 1.5 *U*_eq_(O) using a riding model.

### Crystal data

**Table:**

[Mn(NCSe)_2_(C_12_H_10_N_2_)(CH_4_O)_2_]·C_12_H_10_N_2_	*F*(000) = 694
*M**_r_* = 693.42	*D*_x_ = 1.499 Mg m^−^^3^
Monoclinic, *P*2_1_/*c*	Mo *K*α radiation, λ = 0.71073 Å
Hall symbol: -P 2ybc	Cell parameters from 14170 reflections
*a* = 7.3580 (6) Å	θ = 2.8–25.0°
*b* = 17.2445 (11) Å	µ = 2.83 mm^−^^1^
*c* = 12.1219 (9) Å	*T* = 220 K
β = 92.630 (9)°	Block, yellow
*V* = 1536.5 (2) Å^3^	0.13 × 0.08 × 0.05 mm
*Z* = 2	

### Data collection

**Table:**

Stoe IPDS-1 diffractometer	2633 independent reflections
Radiation source: fine-focus sealed tube	2072 reflections with *I* > 2σ(*I*)
Graphite monochromator	*R*_int_ = 0.091
phi scan	θ_max_ = 25.0°, θ_min_ = 2.8°
Absorption correction: numerical (*X-SHAPE* and *X-RED32*; Stoe & Cie, 2008)	*h* = −8→8
*T*_min_ = 0.754, *T*_max_ = 0.862	*k* = −20→20
14170 measured reflections	*l* = −13→14

### Refinement

**Table:**

Refinement on *F*^2^	Primary atom site location: structure-invariant direct methods
Least-squares matrix: full	Secondary atom site location: difference Fourier map
*R*[*F*^2^ > 2σ(*F*^2^)] = 0.041	Hydrogen site location: inferred from neighbouring sites
*wR*(*F*^2^) = 0.112	H-atom parameters constrained
*S* = 0.99	*w* = 1/[σ^2^(*F*_o_^2^) + (0.077*P*)^2^] where *P* = (*F*_o_^2^ + 2*F*_c_^2^)/3
2633 reflections	(Δ/σ)_max_ < 0.001
179 parameters	Δρ_max_ = 0.38 e Å^−^^3^
0 restraints	Δρ_min_ = −0.78 e Å^−^^3^

### Special details

**Table:**

Geometry. All e.s.d.'s (except the e.s.d. in the dihedral angle between two l.s. planes) are estimated using the full covariance matrix. The cell e.s.d.'s are taken into account individually in the estimation of e.s.d.'s in distances, angles and torsion angles; correlations between e.s.d.'s in cell parameters are only used when they are defined by crystal symmetry. An approximate (isotropic) treatment of cell e.s.d.'s is used for estimating e.s.d.'s involving l.s. planes.
Refinement. Refinement of *F*^2^ against ALL reflections. The weighted *R*-factor *wR* and goodness of fit *S* are based on *F*^2^, conventional *R*-factors *R* are based on *F*, with *F* set to zero for negative *F*^2^. The threshold expression of *F*^2^ > σ(*F*^2^) is used only for calculating *R*-factors(gt) *etc*. and is not relevant to the choice of reflections for refinement. *R*-factors based on *F*^2^ are statistically about twice as large as those based on *F*, and *R*- factors based on ALL data will be even larger.

### Fractional atomic coordinates and isotropic or equivalent isotropic displacement parameters (Å^2^)

**Table:**

	*x*	*y*	*z*	*U*_iso_*/*U*_eq_	
Mn1	1.0000	0.5000	0.5000	0.02859 (18)	
N1	0.8412 (3)	0.59745 (16)	0.5588 (2)	0.0411 (6)	
C1	0.7531 (4)	0.65174 (18)	0.5703 (3)	0.0383 (7)	
Se1	0.61619 (5)	0.73559 (2)	0.58880 (5)	0.0748 (2)	
N10	0.8026 (3)	0.49379 (14)	0.3495 (2)	0.0317 (5)	
C10	0.7810 (4)	0.55472 (17)	0.2815 (3)	0.0337 (7)	
H10	0.8352	0.6020	0.3032	0.040*	
C11	0.6839 (4)	0.55203 (17)	0.1814 (3)	0.0329 (6)	
H11	0.6737	0.5964	0.1366	0.039*	
C12	0.6011 (3)	0.48261 (16)	0.1474 (2)	0.0281 (6)	
C13	0.6159 (4)	0.42049 (17)	0.2201 (3)	0.0342 (7)	
H13	0.5574	0.3733	0.2025	0.041*	
C14	0.7172 (4)	0.42818 (17)	0.3185 (3)	0.0358 (7)	
H14	0.7264	0.3852	0.3661	0.043*	
C15	0.5041 (4)	0.47366 (18)	0.0402 (3)	0.0312 (6)	
H15	0.4423	0.4267	0.0268	0.037*	
N30	0.9261 (4)	0.26379 (17)	0.5841 (4)	0.0604 (9)	
C30	1.0231 (6)	0.2228 (2)	0.6583 (4)	0.0605 (10)	
H30	1.0691	0.2483	0.7222	0.073*	
C31	1.0604 (5)	0.1445 (2)	0.6469 (4)	0.0528 (9)	
H31	1.1297	0.1182	0.7021	0.063*	
C32	0.9951 (4)	0.10541 (19)	0.5537 (3)	0.0413 (8)	
C33	0.8968 (5)	0.1487 (2)	0.4737 (4)	0.0576 (10)	
H33	0.8518	0.1252	0.4081	0.069*	
C34	0.8666 (5)	0.2261 (2)	0.4925 (5)	0.0663 (12)	
H34	0.8004	0.2544	0.4379	0.080*	
C35	1.0281 (4)	0.02177 (19)	0.5422 (3)	0.0403 (7)	
H35	1.0950	−0.0029	0.6001	0.048*	
O1	0.8375 (3)	0.41425 (12)	0.58486 (19)	0.0386 (5)	
H1O1	0.8741	0.3687	0.5840	0.058*	
C2	0.6647 (5)	0.4215 (2)	0.6300 (4)	0.0636 (11)	
H2A	0.6565	0.4712	0.6671	0.095*	
H2B	0.6480	0.3800	0.6827	0.095*	
H2C	0.5709	0.4183	0.5713	0.095*	

### Atomic displacement parameters (Å^2^)

**Table:**

	*U*^11^	*U*^22^	*U*^33^	*U*^12^	*U*^13^	*U*^23^
Mn1	0.0300 (3)	0.0289 (3)	0.0260 (3)	0.0065 (2)	−0.0084 (2)	−0.0038 (2)
N1	0.0433 (14)	0.0367 (14)	0.0427 (17)	0.0107 (12)	−0.0045 (12)	−0.0074 (12)
C1	0.0337 (14)	0.0325 (16)	0.048 (2)	−0.0011 (13)	−0.0026 (13)	−0.0061 (13)
Se1	0.0493 (3)	0.0317 (3)	0.1452 (5)	0.01097 (15)	0.0249 (3)	−0.0019 (2)
N10	0.0335 (12)	0.0324 (13)	0.0282 (13)	0.0039 (10)	−0.0084 (10)	−0.0004 (10)
C10	0.0348 (14)	0.0307 (15)	0.0344 (17)	0.0000 (11)	−0.0100 (12)	−0.0011 (12)
C11	0.0376 (14)	0.0320 (15)	0.0282 (17)	0.0010 (11)	−0.0072 (12)	0.0040 (11)
C12	0.0232 (12)	0.0339 (15)	0.0268 (15)	0.0029 (10)	−0.0045 (10)	0.0019 (11)
C13	0.0350 (14)	0.0332 (16)	0.0337 (17)	−0.0035 (11)	−0.0062 (12)	−0.0011 (12)
C14	0.0410 (16)	0.0329 (16)	0.0324 (17)	0.0011 (12)	−0.0091 (13)	0.0031 (12)
C15	0.0263 (13)	0.0369 (15)	0.0297 (16)	0.0013 (11)	−0.0065 (11)	−0.0009 (12)
N30	0.0468 (17)	0.0362 (17)	0.099 (3)	0.0034 (13)	0.0122 (17)	0.0041 (17)
C30	0.059 (2)	0.046 (2)	0.078 (3)	0.0011 (17)	0.010 (2)	−0.001 (2)
C31	0.0517 (19)	0.045 (2)	0.062 (3)	0.0026 (15)	0.0041 (17)	0.0050 (17)
C32	0.0278 (14)	0.0377 (17)	0.059 (2)	0.0014 (12)	0.0099 (13)	0.0125 (15)
C33	0.0503 (19)	0.0381 (19)	0.083 (3)	0.0021 (15)	−0.0075 (19)	0.0109 (19)
C34	0.050 (2)	0.042 (2)	0.106 (4)	0.0091 (16)	−0.007 (2)	0.020 (2)
C35	0.0299 (14)	0.0361 (17)	0.055 (2)	0.0042 (12)	0.0062 (13)	0.0136 (14)
O1	0.0332 (10)	0.0350 (12)	0.0476 (14)	0.0057 (8)	0.0001 (9)	0.0008 (9)
C2	0.052 (2)	0.055 (2)	0.085 (3)	0.0040 (17)	0.023 (2)	−0.007 (2)

### Geometric parameters (Å, º)

**Table:**

Mn1—N1	2.185 (3)	C15—H15	0.9400
Mn1—N1^i^	2.185 (3)	N30—C30	1.327 (6)
Mn1—O1	2.188 (2)	N30—C34	1.343 (6)
Mn1—O1^i^	2.188 (2)	C30—C31	1.386 (6)
Mn1—N10^i^	2.281 (2)	C30—H30	0.9400
Mn1—N10	2.281 (2)	C31—C32	1.383 (6)
N1—C1	1.151 (4)	C31—H31	0.9400
C1—Se1	1.782 (3)	C32—C33	1.399 (5)
N10—C14	1.340 (4)	C32—C35	1.470 (5)
N10—C10	1.341 (4)	C33—C34	1.374 (6)
C10—C11	1.381 (4)	C33—H33	0.9400
C10—H10	0.9400	C34—H34	0.9400
C11—C12	1.397 (4)	C35—C35^iii^	1.321 (7)
C11—H11	0.9400	C35—H35	0.9400
C12—C13	1.388 (4)	O1—C2	1.413 (4)
C12—C15	1.462 (4)	O1—H1O1	0.8300
C13—C14	1.384 (4)	C2—H2A	0.9700
C13—H13	0.9400	C2—H2B	0.9700
C14—H14	0.9400	C2—H2C	0.9700
C15—C15^ii^	1.331 (6)		
			
N1—Mn1—N1^i^	180.00 (14)	N10—C14—H14	118.3
N1—Mn1—O1	93.12 (10)	C13—C14—H14	118.3
N1^i^—Mn1—O1	86.88 (10)	C15^ii^—C15—C12	125.6 (4)
N1—Mn1—O1^i^	86.88 (10)	C15^ii^—C15—H15	117.2
N1^i^—Mn1—O1^i^	93.12 (10)	C12—C15—H15	117.2
O1—Mn1—O1^i^	180.00 (8)	C30—N30—C34	116.6 (3)
N1—Mn1—N10^i^	91.91 (9)	N30—C30—C31	123.6 (4)
N1^i^—Mn1—N10^i^	88.09 (9)	N30—C30—H30	118.2
O1—Mn1—N10^i^	89.85 (9)	C31—C30—H30	118.2
O1^i^—Mn1—N10^i^	90.15 (9)	C32—C31—C30	119.6 (4)
N1—Mn1—N10	88.09 (9)	C32—C31—H31	120.2
N1^i^—Mn1—N10	91.91 (9)	C30—C31—H31	120.2
O1—Mn1—N10	90.15 (9)	C31—C32—C33	117.0 (3)
O1^i^—Mn1—N10	89.85 (8)	C31—C32—C35	120.2 (3)
N10^i^—Mn1—N10	180.000 (1)	C33—C32—C35	122.8 (4)
C1—N1—Mn1	167.9 (3)	C34—C33—C32	119.1 (4)
N1—C1—Se1	179.6 (3)	C34—C33—H33	120.4
C14—N10—C10	116.6 (2)	C32—C33—H33	120.4
C14—N10—Mn1	122.50 (19)	N30—C34—C33	123.9 (4)
C10—N10—Mn1	120.54 (19)	N30—C34—H34	118.0
N10—C10—C11	123.8 (3)	C33—C34—H34	118.0
N10—C10—H10	118.1	C35^iii^—C35—C32	125.7 (4)
C11—C10—H10	118.1	C35^iii^—C35—H35	117.2
C10—C11—C12	119.3 (3)	C32—C35—H35	117.2
C10—C11—H11	120.3	C2—O1—Mn1	130.0 (2)
C12—C11—H11	120.3	C2—O1—H1O1	112.7
C13—C12—C11	117.0 (3)	Mn1—O1—H1O1	116.7
C13—C12—C15	120.3 (3)	O1—C2—H2A	109.5
C11—C12—C15	122.8 (3)	O1—C2—H2B	109.5
C14—C13—C12	119.8 (3)	H2A—C2—H2B	109.5
C14—C13—H13	120.1	O1—C2—H2C	109.5
C12—C13—H13	120.1	H2A—C2—H2C	109.5
N10—C14—C13	123.4 (3)	H2B—C2—H2C	109.5

Symmetry codes: (i) −*x*+2, −*y*+1, −*z*+1; (ii) −*x*+1, −*y*+1, −*z*; (iii) −*x*+2, −*y*, −*z*+1.

### Hydrogen-bond geometry (Å, º)

**Table:**

*D*—H···*A*	*D*—H	H···*A*	*D*···*A*	*D*—H···*A*
O1—H1*O*1···N30	0.83	1.85	2.675 (4)	173
